# Does Every Subdural Hematoma Patient Need an Embolization?

**DOI:** 10.1007/s00062-024-01425-z

**Published:** 2024-05-16

**Authors:** Jens Fiehler, Matthias Bechstein

**Affiliations:** Department of Diagnostic and Interventional Neuroradiology, University Medical Center Hamburg-Eppendorf, Hamburg, Germany

Chronic subdural hematomas (cSDHs) can lead to significant morbidity and mortality, even with treatment. A major problem is the risk of recurrence after initial surgical evacuation via burr hole or craniectomy. Studies have reported recurrence rates ranging from 5 to 30% after initial treatment [1].

Originally, cSDH recurrence was attributed to a venous re-bleed from primarily ruptured bridging veins into the subdural space. More recently, the re-bleeding from newly formed, fragile arteries has been hypothesized to be the cause of cSDH growth and recurrence. Within hours of the initial venous bleed, the hematoma coagulates. An inflammatory response over the next days leads to a forming a membrane giving rise to a new fragile vessels that continuously exude blood into the subdural space [2, 3]. Stopping the blood supply tips the balance toward resorption and hematoma resolution [4].

Over the last few years, middle meningeal artery embolization (MMAE) emerged as a promising cSDH treatment. MMAE could transform non-acute SDH management, especially in the elderly, potentially surpassing the impact of large vessel stroke on neurointerventional practice. Clinical trials are essential for validation of its efficacy and safety compared with standard management. In recent model calculations, the incidence of SDH was 52/100,000 persons/year surpassing the 32/100,000 persons/year of large vessel occlusions [5].

## What Is New?

Three randomized controlled trials analyzing the effect of embolization of the middle cerebral artery (MMAE) for treatment of chronic subdural hematoma (cSDH) have been presented during the International Stroke Conference in early February. All of them showed clinical superiority of patients treated with MMAE using liquid embolics, either Onyx (Medtronic, Minnesota, USA) or Squid (Balt, Montmorency, France). One of the RCTs (EMBOLISE, NCT04402632) presented only patients with surgical hematoma evacuation with or without additional MMAE. The other two studies presented combined randomized data patients with and without surgical hematoma evacuation (STEM, NCT04410146 and MAGIC-MT, NCT04700345). All three studies reported significantly lower event rates in the treatment arm (Table 1). This is a very strong and robust efficacy signal, considering the heterogeneity of inclusion criteria and of endpoint definitions among the studies.

**Table 1:** Summary of the presented studies

	Key inclusion criteria	Primary outcome	Major results
**EMBOLISE** (craniotomy or burr-hole drainage patients, *n* = 400)	– Moderate or severe cSDH – Motor deficits, – Severe symptoms, – Midline shift ≥ 5 mm or cSDH thickness > 15 mm – Pre-morbid mRS 0‑3	cSDH recurrence or progression requiring surgical treatment within 90 days	Endpoint events – 4.1% with MMA embolization plus standard management – 11.3% with standard management alone RR 0.36 ARR 7.3% NNT 13.8
**MAGIC-MT** (burr-hole drainage patients and non-surgical patients, *n* = 722)	– Symptomatic SDH – < 30% hyperdense, septations – Mass effect (midline shift or brain deformation). – Pre-morbid mRS 0–2	cSDH recurrence or progression requiring surgical treatment within 90 days	Endpoint events – 7.2% with MMA embolization plus standard management – 12.2% with standard management alone RR 0.59 ARR 4.9% NNT 20.3
**STEM** (burr-hole drainage patients and non-surgical patients randomized, *n* = 310)	– Neurological symptoms – cSDH thickness ≥ 10 mm – Pre-morbid mRS 0–1 – cSDH exerts mass effect	Treatment failure within 180 days (any of the following): – Residual or re- SDH accumulation (≥ 10 mm) or – Re-operation, surgical rescue – New, disabling stroke after enrollment, myocardial infarction (MI) or death from neurological cause	Endpoint events – 15.2% with MMA embolization plus standard management – 39.2% with standard management RR 0.39 ARR 23.9% NNT 4.2

## Which Patients Are We Talking About?

All studies included symptomatic patients with chronic subdural hematomas. Symptoms included headache, short-term cognitive decline, speech difficulty or aphasia, gait impairment, focal weakness, sensory deficits, and seizures. The studies did not include asymptomatic cSDHs and no acute SDHs. Most patients enrolled in the studies were randomized for MMAE as adjunct to surgical treatment (burr hole in all studies, also craniotomy in EMBOLISE). In STEM, the positive effect of MMAE was primarily driven by non-surgical patients (19.1% vs 59.2%; *P* = 0.001), while the MMAE effect in patients with additional surgery was not statistically superior to surgery alone (12.3% vs 25.4%, *P* = 0.058) [6].

## How Relevant Are the Primary Outcomes?

The cSDH recurrence or progression requiring surgical treatment within 90 days is a highly relevant clinical outcome. If severe neurological symptoms persist, the decision for re-treatment is straightforward. But it is important to understand that while symptoms may also be mild, unspecific or even absent, the pure existence of a residual or recurrent hematoma cavity complicates clinical management in these often multimorbid patients. This accounts in particular for delayed or mitigated anticoagulation regimens for secondary cardiovascular diseases in order to minimize the risk of acute bleeding into the chronic hematoma [7].

Repeat surgery in the elderly along with inherent immobility and prolonged hospitalization generally constitutes a major risk factor for morbidity and mortality [8]. In relation to a cSDH, this effect is presumably even stronger since surgery of a hematoma in a then membranous and multicavited subdural space often requires a larger more invasive craniotomy for optimized removal of all components [9, 10].

Some studies estimate the treatment costs for recurrent cSDHs up to 132% higher than the treatment costs of non-recurrent cSDHs [11]. Besides longer hospitalization, this effect is likely also due to more frequent outpatient follow-up with CT. This constitutes a significant health economical challenge. Reducing recurrences is important not only for neurological improvement, but also for reducing secondary complications in a particular vulnerable patient population, and ultimately also health care costs. The results of the three randomized controlled trials point to a powerful new tool for mitigating the recurrence risk.

Further analyses are needed to get a better picture of who is at particular risk of recurrence and benefits most from MMAE. Surgery and MMAE should not be seen as competing but rather complementary procedures. MMAE will likely never be as effective as surgery for immediate reduction of a large space occupying hematoma. MMAE is aimed at interrupting the vicious circle of repeated secondary bleeding from fragile vessels of the subdural membranes, primarily in smaller cSDHs and secondarily after surgical reduction or removal of a larger cSDH.

Decision making in treatment of cSDH patients will always rely on both clinical symptoms and imaging, sometimes requiring careful balancing. Clinical assessment may be difficult because of other disease conditions or the unspecific nature of symptoms. Radiological assessment may also be compromised because methods of hematoma measurement vary [12]. The primary outcome parameter “recurrence or progression requiring surgical treatment” is a result of this balanced decision making and should also be used in future studies.

## Conclusion

Treating symptomatic cSDHs with MMAE using liquid embolic agents will become a standard procedure. Likely the number of MMAE procedures will reach or even surpass the number of thrombectomies.
